# CO_2_ Capture in Biocompatible Amino Acid Ionic Liquids: Exploring the Reaction Mechanisms for Bimolecular Absorption Processes

**DOI:** 10.1021/acs.jpcb.1c02945

**Published:** 2021-05-19

**Authors:** Stefano Onofri, Enrico Bodo

**Affiliations:** † Department of Chemistry, University of Rome “La Sapienza”, Piazzale A. Moro 5, 00185 Rome, Italy; ‡ Multi-Scale Mechanics (MSM), Thermal and Fluid Engineering, Faculty of Engineering Technology, University of Twente, 7500 AE, Enschede, The Netherlands

## Abstract

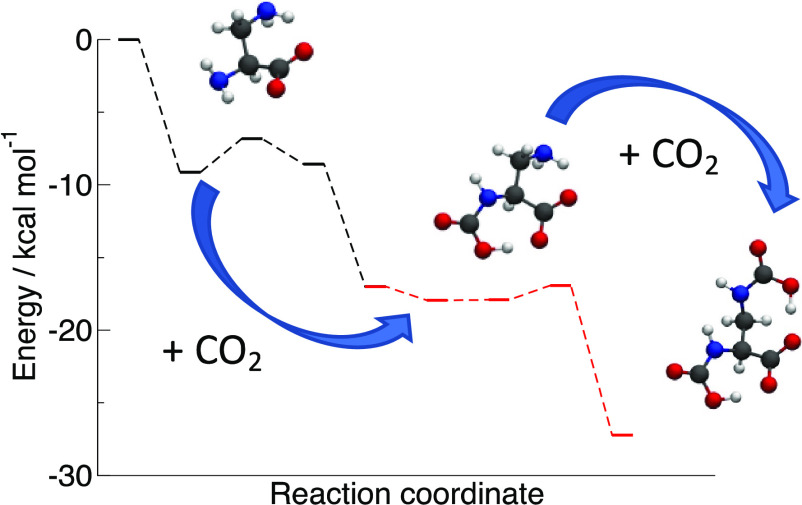

CO_2_ capture at the production site represents one of the accessible ways to reduce its emission in the atmosphere. In this context, CO_2_ chemisorption is particularly advantageous and is often based on exploiting a liquid containing amino groups that can trap CO_2_ due to their propensity to react with it to yield carbamic derivatives. A well-known class of ionic liquids based on amino acid anions might represent an ideal medium for CO_2_ capture because, at difference with present implementations, they are known to be fully biocompatible. One of the problems is however the relatively low molar ratio of CO_2_ absorption. Increasing this ratio turns out to be possible by choosing appropriate anions. We present here a set of accurate computations to elucidate the possible reaction paths that allow the anion to absorb two CO_2_ molecules, thus effectively doubling the overall intake. An extensive exploration of some reaction mechanisms suggests that some of them might be quite efficient even under mild conditions.

## 1 Introduction

The increase in CO_2_ emission by anthropic activities represents one of the most serious threats for the ecosystem as it represents the main source of worldwide temperature increase.^1−3^ Since the majority of CO_2_ emissions are due to fossil fuels processing^4^ and, specifically, more than half of these come from power plants, part of the overall research effort has been devoted to finding economic and environmentally friendly ways to capture and remove CO_2_ from flue gases at the production sites.^5−7^ One of the present capture techniques is based on chemisorption and, in particular, on exploiting the chemical reaction of CO_2_ with amines.^8,9^ These technologies, however, are currently based on corrosive and harmful aqueous amine solutions and represent expensive and non-eco-friendly approaches.^10^

Ionic liquids (ILs) have been proposed as an alternative to aqueous amine solutions for CO_2_ capture since the early years of the past decade^11^ and the study of these task-specific ILs is, at the moment, a very active field of research recently summarized in previous reviews.^12−15^ The typical advantage of ILs over other solvents lies in their negligible vapor pressure and in their tunable chemical composition, which allows them to be optimized for specific tasks.^16,17^ CO_2_ absorption by ILs can be achieved by both physisorption and chemisorption, with the latter often having a greater efficiency. CO_2_ chemisorption can be realized by inserting amino groups in their molecular components to allow their reaction with CO_2_ to form carbamates or carbamic acids.^18−23^

Among the ILs specifically synthesized for CO_2_ chemisorption, those based on anions made by a deprotonated amino acid (AA)^24,25^ seem to yield a positive balance between absorption capacity, synthesis cost, and biocompatibility.^26−28^ In these ILs, CO_2_ absorption occurs to various extents depending on the physical conditions and on their molecular components, from 0.5 mol of CO_2_ per mol of IL (1:2 mechanism) to 1 mol of CO_2_ per 1 mol of IL (1:1 mechanism) and to even higher molar fractions (2:1 mechanisms).^21,22,29,30^

**Scheme 1:**
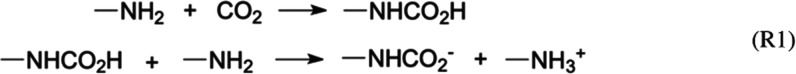
General Reaction Process of CO_2_ with the −NH_2_ Group

The general reaction scheme of CO_2_ with amines is known^31,32^ and consists of the two-reactions process (R1) reported in Scheme 1. If the second reaction takes place, the absorption process is typically characterized by an overall 1:2 stoichiometry and by a low efficiency since two AA anions are used to incorporate only one CO_2_ molecule. Instead, if the second reaction is inefficient or hindered, the overall absorption process proceeds with a 1:1 stoichiometry. If a second attack by another CO_2_ molecule is possible (either on the residual NH group of the AA anion, or on another NH_2_ group of the same anion), the final stoichiometry tends to a 2:1 molar ratio. It is obvious that, to promote efficiency, it would be desirable to reach the latter situation.

**Scheme 2:**
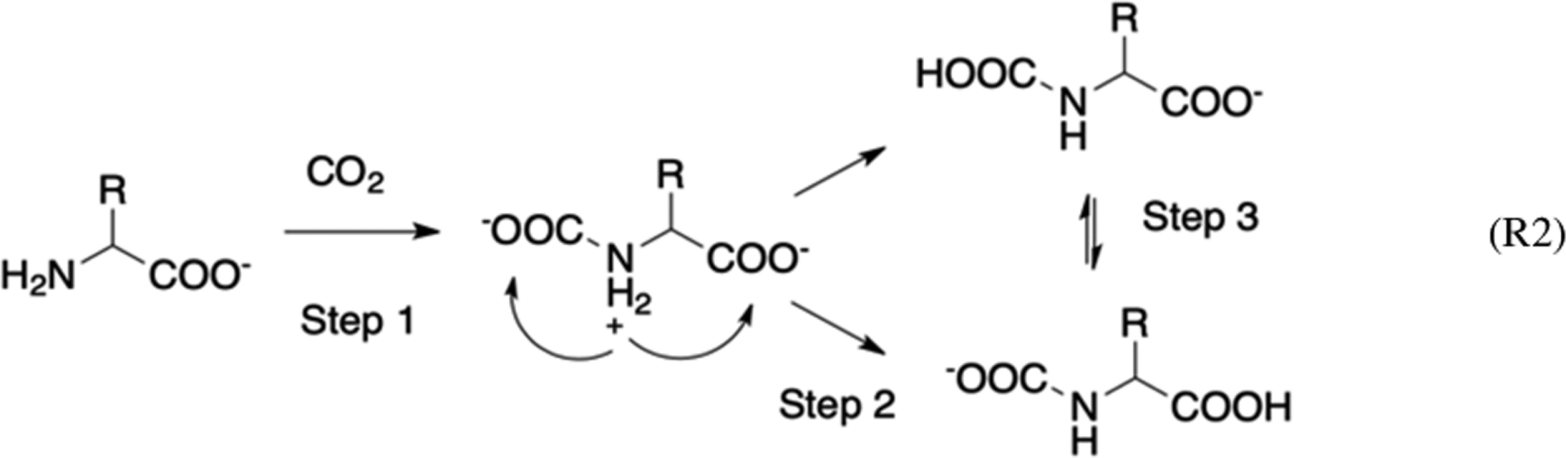
General Reaction between an AA Anion and a First Molecule of CO_2_ In the first step, a zwitterionic pre-reaction complex is formed. A subsequent PT (step 2) removes the zwitterion and forms a carbamic acid derivative or a carbamate depending on the preferential site of PT. An isomerization equilibrium can then take place (step 3).

The CO_2_ addition (first reaction of R1) can be further divided into the three steps^33^ shown in Scheme 2: the initial one is the formation of a pre-reaction complex with zwitterionic character followed, in step 2, by a proton transfer (PT) from the positive nitrogen to one of the carboxylates. The final product is represented by the most stable of the two possible tautomeric forms, which can interconvert via further inter- or intramolecular PTs (step 3).

Several computational descriptions of this mechanism have appeared in the literature^34−39^ and have been summarized in a review by Sheridan et al.^40^ More recently, we have explored the absorption mechanisms of a single CO_2_ molecule by different prototypical AA anions.^32^ Overall, the features of R2 can be summarized as follows:

1. The efficiency of formation of the pre-reaction complex is limited by the diffusion of CO_2_ in the liquid and by the energy necessary to “desolvate” the amino group and make it available for the reaction. The latter can be considered as the energy required to break the ionic couple in which the anion is bound.^35^ This explains why the high viscosity of these fluids is an issue and, at the moment, a key factor that severely limits their practical usability.^41,42^
2. Once the CO_2_ molecule is able to attack the −NH_2_ group, the ensuing reaction toward the carbamic AA derivative is almost invariably exothermic, hence thermodynamically favored. The rate-limiting step is the PT from the nitrogen to the carboxylate that forms the AA anion carbamic derivative. Owing to interionic Coulomb repulsion, the PT is more likely to occur within the same molecule rather than between two different anions. In principle, the kinetics of the reaction is therefore determined by the energy barrier along the PT step, but we have recently shown that, depending on the nature of the AA, there exist reactive pathways with null or negligible activation barriers.^32^
3. The formation of the primary AA carbamic derivative can be followed by additional isomerization reactions to form other isomers, depending on which structure is more stable.

One of the problems that has been only seldom explored in previous works^43,44^ is why some of the liquids based on AA anions present molar absorption intakes (2:1 mechanism), which are larger than one. On the one hand, it is obvious that AA anions such as [Lys]^−^ that have two −NH_2_ groups allow for molar intakes larger than one thanks to the double reaction sites (for [Lys]^−^, a molar ratio of 1.6 has been measured^43,45^). On the other hand, ILs based on simple AA anions such as [Gly]^−^ (with a molar ratio of 1.2^46^) or doubly deprotonated ones such as [Asp]^2–^ (with molar ratio ∼2^43^) clearly present the ability to incorporate, at least partially, a second CO_2_ molecule, albeit in a less obvious way.

**Scheme 3:**
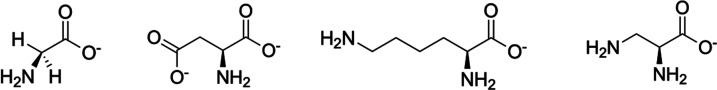
Structures of the 4 AA Anions Used in This Work: From Left to Right, Glycinate, Doubly Deprotonated Aspartate, Lysinate, and 2,3-Diaminopropionate

It is the purpose of this work to explore via ab initio calculations the mechanism at the basis of these experimental results using four different prototypical AA anions: [Gly]^−^, [Asp]^2–^, [Lys]^−^, and [DAP]^−^ (2,3-diaminopropionate), a smaller analogue of [Lys]^−^. Their structures are shown in Scheme 3.

To maintain generality, we have to simplify the overall problem to make the results independent of the many variables at play. First of all, in the framework of ab initio calculations, we are unable to account for the presence of an explicit surrounding liquid. In ref (32), we have taken into account the environmental effects using the polarizable continuum model (PCM) approximation using the parametrization for a solvent with a medium dielectric constant.^35^ The results indicate that the presence of such environment does not alter significantly the overall reaction profiles with only minor variations of the PT barrier. The main effect of the surrounding dielectric medium is a reduction of the reactant-to-product energy difference (Δ*H* of reaction). This is simply due to the condensation nature (A + B → C) of the present reactions. A dielectric medium induces a greater stabilization of the bimolecular reactants than that of the final product because of the appearance of two solvation shells in the A + B channel. Given this situation, we have decided here to focus our discussion mainly on gas-phase results, but we will present also continuum model solvation results for reference.

To make our calculations independent of the peculiarities of the actual liquid and to provide a general mechanism, we will not include a cationic partner. While the nature of the cation in the reaction has been proven, under certain conditions, to be relevant,^30,47^ to correctly address its role within the present context and within our computational approach is not easy. In an approach like ours where the system is isolated, the binding motif and energy of the cation can be arguably very different from those in the actual bulk phase of ILs. In other words, apart from the case in which the cation partakes to the absorption reaction because it possesses amino groups, its role can emerge because it influences the structural and frictional properties of the fluid, e.g., the diffusion of CO_2_ in it. Such many-body effects are however precluded to our present investigation and, it would be difficult to ascertain the peculiar effects due to cations. In addition, the study reported by Shaikh et al.^39^ indicates how, using a computational approach based on isolated ionic couples, the effect of different weakly coordinating cations (such as those typically used in ILs) can be relatively unimportant in modifying the reaction profile of the [Gly]^−^ anion (compare Figures 2 and 3 of the cited work). That the effect of the cation choice on the reaction profile can be small is also seen in the rather extensive data reported by Firaha and Kirchner^35^ and, in particular, in the reported energies associated with the proton transfer step (Table S2 in the cited paper) that show significant variations among the AA anions but change only slightly for different cations.

Finally, the study of the influence of the cationic partner on the reaction mechanisms would require a complete, systematic study of its role as a function of its coordinating properties, size, and steric shape. This task, albeit worth undertaking, lies well outside the scope of the present treatment, which aims at the characterization of the reaction profile of the unperturbed anions. We believe that the exploration of the reaction mechanisms of the anions alone (an approach that has its limits but is at least universal for all AA-based ILs) represents a prerequisite for further studies: on the one hand, our simplified approach eases the interpretation of the peculiar experimental evidence for specific ILs and, on the other, helps in setting the starting point for possible future theoretical studies in more specific conditions.

## 2 Computational Methods

The ab initio calculations have been carried out for the isolated reagents, i.e., the AA anion and the isolated CO_2_ (**R**), for the pre-reaction complex (**CR**) and for the products (**P**). Since the formation of **CR** from **R** is barrierless, the dominant transition state (**TS**) has been localized between **CR** and **P**. When the product **P** presents more than one tautomeric or isomeric structure, we will report only the one with the lowest energy. For each structure, we have performed an unconstrained optimization and evaluated the harmonic frequencies using the dispersion-corrected B3LYP-D3 functional^48^ with the 6-311+G(d,p) basis set. All minima and saddle points have been verified by computing the full hessian and by checking the relative vibrational frequencies. When needed, we have made sure the uniqueness of the transition state by computing the intrinsic reaction coordinate (IRC), but we will not report these consistency-check calculations. The computational model has been previously verified as accurate enough when compared to CBS-QB3 and G4MP2 composite methods.^32^ The Gaussian16^49^ package was used for all the ab initio calculations. We have included the zero-point-energies in all of the energetic values reported in the rest of the paper. Regarding the harmonic analysis, we should point out that, even though it provides a value for the Gibbs free energy, its calculation is based on the perfect gas model and cannot be considered as entirely accurate for a liquid environment where the rotational and translational degrees of freedom are hindered.

The presence of a surrounding medium has been evaluated by repeating the calculations in a continuum SMD (solvent model density)^50^ environment with the parameters of acetonitrile, which has a dielectric constant of 35, which is only slightly greater than that of AA-based ILs.^51^ This choice should ensure that we are including in our calculations a sufficient dielectric screening to induce a stabilization of charge-separated species and mimicking the dielectric response (albeit in slight excess) of the surrounding IL.

## 3 Results and Discussion

### 3.1 Prototype: The Glycinate Anion

We have already presented the mechanism of the reaction of glycinate with the first CO_2_ molecule in ref (32). The initial zwitterionic complex can evolve through two possible pathways that lead to the formation of the carbamic derivative. The proton transfer transition states are characterized by a cyclic structure with four or five atoms in the ring. Only the one with a five-membered ring (owing to the minor strain) is viable and has a low barrier with respect to the zwitterionic complex (2.2 kcal/mol in terms of free energy). This pathway is certainly the one that allows the absorption of the first CO_2_ molecule. The resulting carbamate derivative of the AA anion is now the reagent **R** of the reaction with the second CO_2_ molecule.

**Figure 1:**
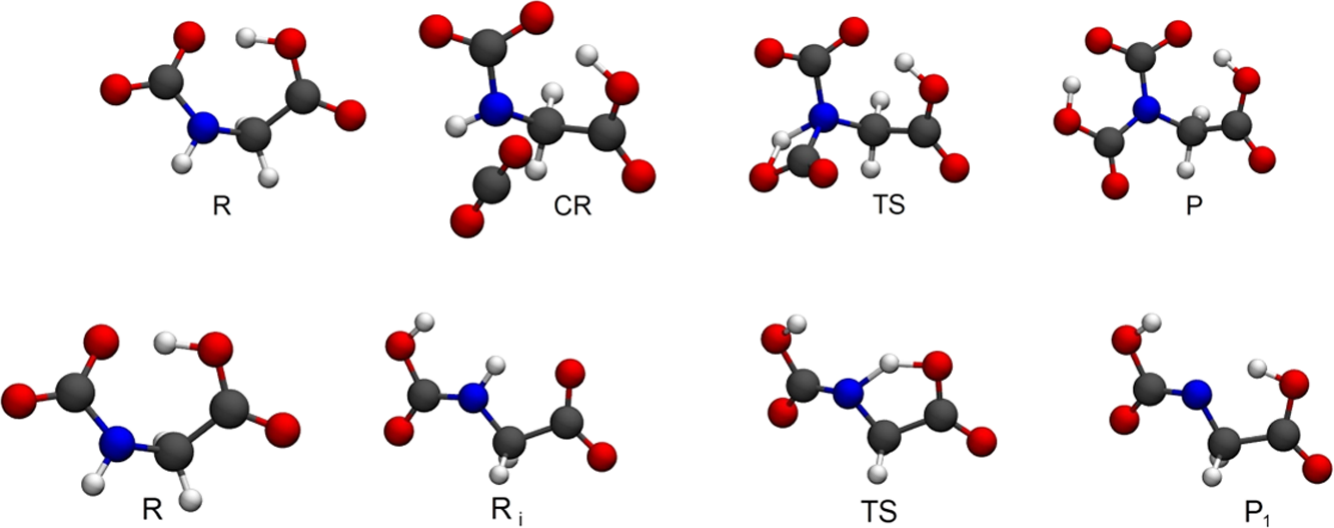
Geometries involved in the addition of a second CO_2_ molecule to the glycinate anion along the PT2-4 path (top) and PT2-i one (bottom). The final reaction of **P**_**1**_ with CO_2_ is not shown, but it directly leads to compound **P** on top.

The first route toward the second CO_2_ molecule absorption (PT2-4) is described by the geometries of the stationary points reported in the top sequence of Figure 1 and by the corresponding energies in Table 1. The initial reagent is the glycinate carbamate derivative **R**, in Figure 1 that is the most stable tautomer of this anion. It shows only a small propensity to bind a second CO_2_ molecule that, in this case, forms only a relatively weak complex (**CR**) with ∼5 kcal/mol of binding energy. The O_2_C–N distance in the weak pre-reaction complex is 2.8 Å and no charge transfer has been detected with the CO_2_ remaining neutral. The insertion of CO_2_ into the molecule costs energy and takes place simultaneously to the proton transfer from the nitrogen to the newly formed carboxyl (**TS**). The cycle has four members and is heavily strained with a C–N–H angle of 70°; hence, its energy is ∼40 kcal/mol above **CR**. This path is clearly completely precluded at low temperatures.

**Table 1:** Energy Differences (at 300 K) for the Insertion of a Second CO_2_ Molecule in the Glycinate Anion through the PT2-4 and PT2-i Mechanisms in kcal/mol^a^

step	Δ(E + ZPE)	Δ*H*	Δ*G*
Mechanism PT2-4			
**R** → **P**	–12.22 (−5.23)	–13.05 (−6.09)	–1.60 (+5.50)
**CR** → **TS**	+39.45 (+41.43)	+38.45 (+40.43)	+42.98 (+43.48)
Mechanism PT2-i			
**R** → **[P**_**1**_**]** → **P**	–12.22 (−5.23)	–13.05 (−6.09)	–1.60 (+5.50)
**R** → **R**_**i**_	+9.03 (+6.14)	+9.50 (+6.63)	+7.99 (+5.17)
**R**_**i**_ → **TS** ∼ **[P**_**1**_**]**	+5.44 (+10.75)	+5.01 (+10.30)	+6.10 (+11.34)

a The values in parentheses have been obtained using the SMD solvation model.

A second reactive path is available to [Gly]^−^ that does not involve a CO_2_–AA complex, but requires a low-energy isomerization of the carbamic derivative. This path, called PT2-i, is illustrated in the bottom sequence of Figure 1, and the energies of the overall reaction are reported in Table 1. The starting point is the same of PT2-4, i.e., the glycinate carbamate derivative **R**. It isomerizes to **R**_i_ that is a carbamic acid. The latter, in turn, passes through a cyclic transition state (**TS**) and isomerizes again into the structure **P**_**1**_, which has a negatively charged (−0.5e) −N– group. The isomerization from **R**_i_ to **P_1_** costs ∼15 kcal/mol overall (∼16 kcal/mol in the solvent model) and the energies of **TS** and **P_1_** are essentially the same within ca. 0.1–0.3 kcal/mol. Compound **P**_**1**_ is a short-lived intermediate whose final reaction with the CO_2_ molecule (not shown) is barrierless and exoergic and leads to the same final compound **P** of PT1-4.

Both the mechanisms PT2-4 and PT2-i are globally exoergic/exothermic of about 12–13 kcal/mol, a value which is reduced in a solvent model to ∼6 kcal/mol because of the greater stabilization of the reactants **R** (two individually solvated molecules) with respect to the product **P** (one molecule). The entropic contribution in passing from two molecule to one is obviously negative and reduces the overall free energy gain of the reaction to −1.6 kcal/mol in vacuo and to +5.5 kcal/mol in a solvent model, but, as we mentioned above, the entropy contribution could be overestimated due to being computed in the perfect gas approximation. Kinetically, PT2-i is far more efficient than PT2-4 since the former has an overall barrier of about 14 kcal/mol (instead of ∼40), which is not prohibitive even at room temperature. The appearance of the PT2-i mechanism in liquids containing the glycinate anion might explain why the overall CO_2_ intake exceeds 1.

### 3.2 Multiple Amino Groups: Lys and DAP

A double intake of CO_2_ in AA-based ILs can simply take place because the anion has two amino groups available for attack by CO_2_. Both [Lys]^−^ and [DAP]^−^ have this kind of structure.

We begin by looking at the DAP anion, which is an AA with a −CH_2_NH_2_ side chain. The reaction with the first CO_2_ molecule takes place following the mechanisms detailed in our previous work^32^ and illustrated in Scheme 2. The first option (PT1-5) sees the CO_2_ attack on the NH_2_ of the AA, and the second one (PT1-6) has the CO_2_ attacking the NH_2_ group of the side chain. This obviously leads to two different final products.

In PT1-5, the reaction is activated by the initial formation of the zwitterionic complex **CR** (see Scheme 2), where the O_2_C–N distance is 1.7 Å, the charge of the NH_2_ group is +0.3e, and the charge of CO_2_ is −0.4e. The **TS** structure is characterized by a five-membered cycle (see Table 2) where the N–H and H–O distances are 1.3 and 1.2 Å, respectively. The proton transfer produces a carbamate that evolves toward the final product **P**, which is a carbamic acid derivative with an intramolecular O–H–O hydrogen bond with an O–O distance of 2.5 Å.

**Table 2:**
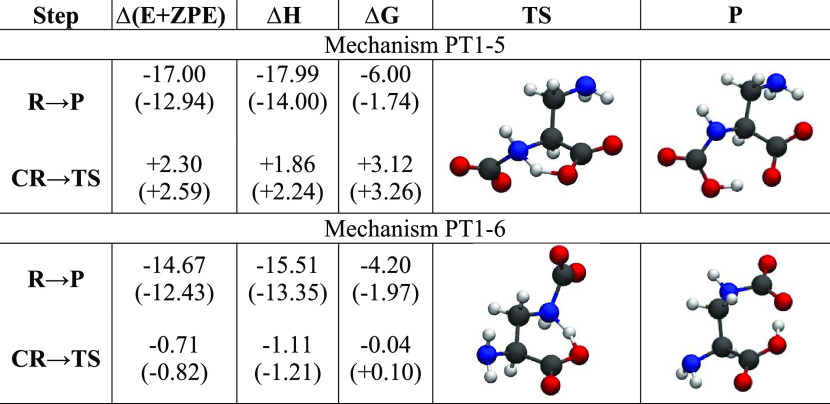
Energy Differences (at 300 K) for the [DAP]^−^ Reaction with the First CO_2_ Molecule along the PT1-5 and PT1-6 Mechanisms in kcal/mol^a^

a The transition state and product structures are also shown. The values in parentheses come from the inclusion of a solvent model.

Analogously, the PT1-6 mechanism initiates from a zwitterionic complex **CR** with an O_2_C–N distance of 1.7 Å, a +0.2e charge on NH_2_, and a −0.5e charge on CO_2_. The **TS** structure is characterized by a six-membered cycle (see Table 2), where the N–H and H–O distances are 1.3 and 1.2 Å, respectively. The final product in this mechanism is directly the carbamate anion derivative **P**.

The overall energetic balance and reaction barriers are summarized in Table 2. The reactions are exothermic/exoergic and proceed with a small activation barrier of 2–3 kcal/mol for PT1-5 and no barrier for PT1-6. Both processes are very effective in incorporating CO_2_ in the liquid. The inclusion of solvent effects does not change much this picture: exothermicity is slightly reduced, but the activation barrier is substantially unaffected.

**Figure 2:**
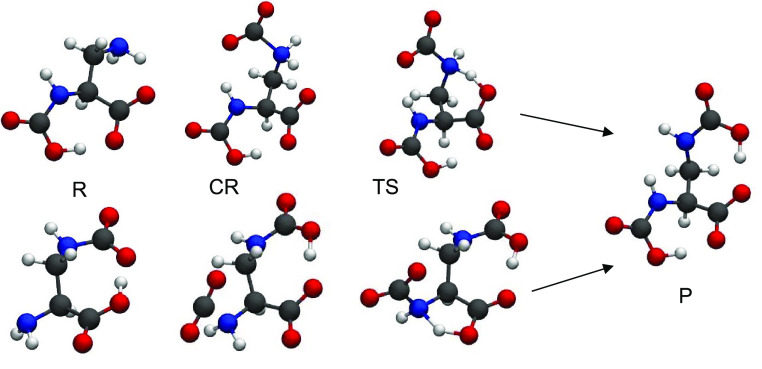
Geometries involved in the addition of a second CO_2_ molecule to the DAP anion. Top sequence: PT2-6, bottom sequence: PT2-5.

Starting from the two structures reported in Table 2 on the right, we have analyzed the two possible further attacks of CO_2_ on the surviving NH_2_ groups. The sequences of structures are reported in Figure 2. The top sequence (PT2-6) begins with the product of PT1-5 and evolves through an initial zwitterionic complex (**CR**) and a six-membered **TS** toward the final **P** product. The **CR** complex is characterized by an O_2_C–N distance of 1.7 Å, a net charge of +0.2 on NH_2_, and a −0.4e charge on CO_2_. The transition state is a six-membered ring with N–H and O–H distances of 1.3 and 1.2 Å, respectively. The final product is an anion with two protonated carbamic acid groups.

The bottom sequence (PT2-5) starts with the product of PT1-6, evolves with a weakly bound **CR** complex, passes through a five-membered ring **TS**, and yields the same product **P**. The pre-reaction complex has no zwitterionic character with a neutral CO_2_ molecule weakly bound to the NH_2_ at 2.7 Å. The transition state has a five-membered ring with N–H and O–H distances in line with the previously described analogous structures.

The energetic features of the PT2-5 and PT2-6 pathways are summarized in Table 3. The PT2-5 mechanism is exothermic/exoergic and remains so in terms of free energy despite the unfavorable entropy changes due to passing from two to one molecule. A barrier of about ∼10 kcal/mol is present and affects the PT step, but the process is nevertheless possible at room temperature. The PT2-6 pathway is also endothermic and not hindered by a significant activation barrier. The latter seems to appear as a very efficient route for the insertion of the second CO_2_ molecule. The calculations including the solvent effect further confirm the existence of this second exothermic pathway with no activation barrier (PT2-6).

**Table 3:** Energy Differences (at 300 K) in kcal/mol for the Second CO_2_ Molecule Insertion in [DAP]^−^ along the PT2-5 and PT2-6 Mechanisms (See Figure 2)^a^

step	Δ(E + ZPE)	Δ*H*	Δ*G*
Mechanism PT2-5			
**R** → **P**	–12.55 (−9.93)	–13.53 (−10.95)	–1.35 (+1.31)
**CR** → **TS**	+9.85 (+8.36)	+8.55 (+7.00)	+13.62 (+11.07)
Mechanism PT2-6			
**R** → **P**	–10.22 (−9.42)	–11.04 (−10.30)	+0.45 (+1.12)
**CR** → **TS**	+0.03 (+1.62)	–0.5 (+1.17)	+1.08 (+2.55)

a The values in parentheses have been obtained using the SMD solvation model.

The lysinate anion behaves in a somewhat analogous way to [DAP]^−^. The side chain is much longer having four methylene groups. The first CO_2_ molecule inserts itself in an analogous way as in [DAP]^−^. We have identified two viable mechanisms which resemble the PT1-5/6 ones for [DAP]^−^. They are both illustrated in Table 4. In PT1-5, the attack is on the AA NH_2_ group and the **TS** has a five-membered ring, while in PT1-9, the attack is on the NH_2_ of the side chain with a **TS** with a nine-membered ring. Both mechanisms begin with the formation of a zwitterionic **CR** complex, which shares the same features of the one we have seen for [DAP]^−^. The only relevant difference with DAP is that the PT1-9 pathway has a “late” transition state where the proton is already near the oxygen with the O–H distance being 1.1 Å and the N–H distance being 1.4 Å.

**Table 4:**
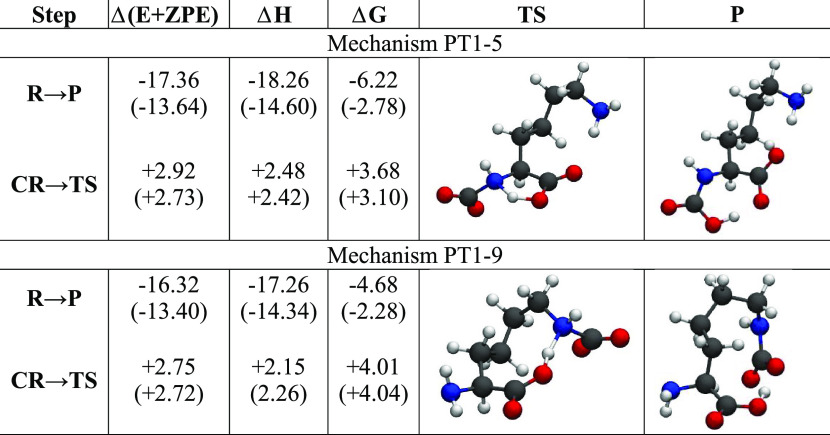
Energy Differences (at 300 K) for the [Lys]^−^ Reaction with the First CO_2_ Molecule along the PT1-5 and PT1-9 Mechanisms in kcal/mol^a^

a The transition state and product structures are also shown. The values in parentheses have been obtained using the SMD solvation model.

Both reactions are exothermic both in vacuo and in the solvent model and are characterized by low activation barriers (∼4 kcal/mol of free energy). Once again, the presence of a solvent medium manifests itself through an overall reduction of the exothermicity of the reaction but has little or no effects on the activation barrier of the process.

**Figure 3:**
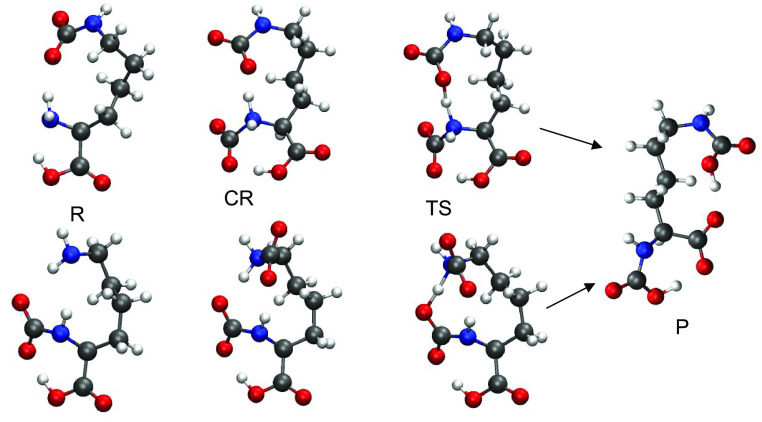
Geometries involved in the addition of a second CO_2_ molecule to the carbamate derivative of the Lys anion. Top sequence: PT2-10a; bottom sequence: PT2-10b.

The two products from PT1-5 and PT1-9 are different molecules that can react with a second CO_2_ molecule. The molecule resulting from the PT1-9 can undergo a second attack on the AA amino group and incorporate the second CO_2_ molecule in as much the same way as in the PT2-5 mechanisms of [DAP]^−^. The PT transfer is, as usual, the rate-determining step in this reaction: it can involve either the nearest carboxyl or the farthest one. It turns out that, in analogy with PT2-5 for [DAP]^−^, the former has a sizable activation barrier (ca. 6–7 kcal/mol), while the latter has a negligible one. This mechanism (PT2-10a) is therefore particularly efficient since it is also exothermic of about ∼18 kcal/mol and is illustrated in Figure 3 (top sequence). The geometric features of this mechanism are not dissimilar from those already described for [DAP]^−^, and we will not repeat them here.

The carbamate derivative of lysinate coming from PT1-5 can also undergo a second CO_2_ addition at the NH_2_ on the side chain through a mechanism that we call PT2-10b and is reported in Figure 3 (bottom sequence). The final product is the same as that produced by the PT2-10a path. This path is however hindered by a very large activation barrier of ∼35 kcal/mol and is therefore highly inefficient.

### 3.3 Doubly Deprotonated AA: The Dianion of Asp

Ionic liquids based on the doubly deprotonated aspartate anion have been found to absorb CO_2_ with a 2:1 mechanism. The first molecule of CO_2_ enters via the two mechanisms PT1-5 and PT1-6 seen for other AA anions and reported by us in ref (32). These paths resemble the two found for the DAP anion, but in this case, the final product is only one because there is only one NH_2_ group. The details and relevant geometries are in Table 5. Both reactions are exothermic/exoergic, and the PT1-6 path has a negligible barrier (ca. 0–2 kcal/mol) and hence, the high absorption efficiency of this anion. The PT1-5 and PT1-6 mechanisms begin with the formation of the same zwitterionic complexes **CR** that is characterized by an O_2_C–N distance of 1.6 Å. The zwitterionic character is here enhanced with respect to the previous cases with a charge of +0.4 on NH_2_ and −0.6 on CO_2_. The final product **P** is a carbamate derivative with the residual proton on the amino acid carboxylate. The structure is stabilized by a strong intramolecular hydrogen bond with an O–O distance of 1.4 Å.

**Table 5:**
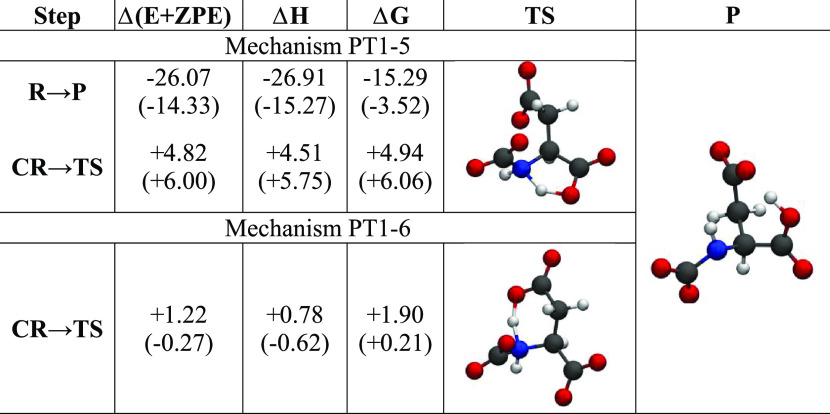
Energy Differences (at 300 K) for the Asp^2–^ Reaction with the First CO_2_ Molecule along the PT1-5 and PT1-6 Mechanisms in kcal/mol^a^

a The transition state and product structures are also shown. The values in parentheses have been obtained using the SMD solvation model.

A second molecule of CO_2_ can also be inserted into the resulting carbamate (**P** in Table 5) exploiting the PT2-4 mechanism that we have already seen for the glycinate anion that involves a four-membered ring proton transfer. As for glycinate, however, the PT2-4 mechanism is hindered by an ∼30 kcal/mol barrier, which renders it impossible at room temperature. The additional flexibility of the [Asp]^2–^ anion with respect to glycinate allows for two more paths to incorporate the second CO_2_ molecule. We have found two very similar mechanisms that differ for the number of atoms involved in the ring of the cyclic transition state during PT. The energetic and the relevant structures are reported in Table 6. For both mechanisms, the starting reagent is the **P** molecule of Table 5, and the final product is the same. The reaction is exothermic albeit only slightly in a solvent medium. Both mechanisms have average to low activation barriers of less than 10 kcal/mol (in terms of free energy) and justify the ability of this anion to bind two CO_2_ molecules. The geometric features of the structures involved (such as N–H and O–H distances in **TS**) are not dissimilar from what has been previously found for the other ions.

**Table 6:**
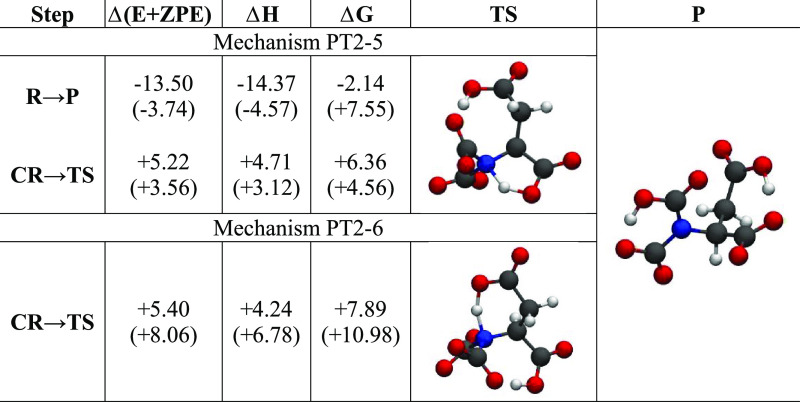
Energy Differences (at 300 K) for the [Asp]^2–^ Reaction with the Second CO_2_ Molecule along the PT2-5 and PT2-6 Mechanisms in kcal/mol^a^

a The transition state and product structures are also shown. The values in parentheses have been obtained using the SMD solvation model.

## 4 Conclusions

In this paper, we have examined the possible absorption mechanisms of two CO_2_ molecules by a selected set of prototypic AA anions. The calculations (in vacuo) show that for all of the anions, there exist possible mechanisms that are viable at room temperature for a double intake of CO_2_.

The first CO_2_ molecule is incorporated in the anion through a reaction with the −NH_2_ group that transforms the AA anion into a carbamic derivative. AA anions with an additional −NH_2_ group can easily react with a second CO_2_ molecule, and we have shown that both lysinate and diaminopropionate present favorable reaction schemes that lead to an efficient double-molar intake of CO_2_, with substantially barrierless reaction profiles.

In other AA anions, there is only a −NH_2_ group and the second molecule has to react with the residual NH group, which is, however, less active toward CO_2_.

This second reaction for the simplest of the AA anion (glycinate) has to proceed through a sequence of isomerization reactions with a substantial energetic cost (15 kcal/mol). The reaction is nevertheless possible, thereby providing a justification of the rather surprising molar intake measured for some ILs based on this simple anion.

The doubly deprotonated aspartate anion has greater conformation mobility than glycinate and the second CO_2_ molecule can be incorporated slightly more easily in the anion through a process, which has a low activation barrier of about 6–7 kcal/mol.

In conclusion, we have shown that different AA anions do provide an efficient and effective absorbent of CO_2_ molecules with sufficiently high molar intakes and that optimization of the environmental conditions such as counterion, temperature, and viscosity might further increase the overall CO_2_ intake to a degree, which might be considered for practical applications.

In principle, the reactions explored here are reversible and could lead to the decarboxylation of the carbamates, hence in CO_2_ desorption from the liquid. In general, the absorption of the first CO_2_ molecule is largely exothermic so that the decarboxylation of the carbamate would be thermodynamically hindered by about 20 kcal/mol for AA anions such as Gly, Lys, and DAP and by even more for Asp. The reactions for the absorption of the second CO_2_ molecule, however, are less exothermic for most of the mechanisms considered here with Δ*H* typically around −12/–15 kcal/mol. It thus turns out that the products emerging from the intake of the second CO_2_ molecule are less stable toward decarboxylation compared to those arising from the absorption of the first one.
